# Piecing Together the Maternal Death Puzzle through Narratives: The Three Delays Model Revisited

**DOI:** 10.1371/journal.pone.0052090

**Published:** 2012-12-19

**Authors:** Viva Combs Thorsen, Johanne Sundby, Address Malata

**Affiliations:** 1 Department of Community Medicine, University of Oslo, Oslo, Norway; 2 Department of Maternal and Child Health, Kamuzu College of Nursing, University of Malawi, Lilongwe, Malawi; Institute of Tropical Medicine Antwerp, Belgium

## Abstract

**Background:**

In Malawi maternal mortality continues to be a major public health challenge. Going beyond the numbers to form a more complete view of why women die is critical to improving access to and quality of emergency obstetric care. The objective of the current study was to identify the socio-cultural and facility-based factors that contributed to maternal deaths in the district of Lilongwe, Malawi.

**Methods:**

Retrospectively, 32 maternal death cases that occurred between January 1, 2011 and June 30, 2011 were reviewed independently by two gynecologists/obstetricians. Interviews were conducted with healthcare staff, family members, neighbors, and traditional birth attendants. Guided by the grounded theory approach, interview transcripts were analyzed manually and continuously. Emerging, recurring themes were identified and excerpts from the transcripts were categorized according to the Three Delays Model (3Ds).

**Results:**

Sixteen deaths were due to direct obstetric complications, sepsis and hemorrhage being most common. Sixteen deaths were due to indirect causes with the main cause being anemia, followed by HIV and heart disease. Lack of recognizing signs, symptoms, and severity of the situation; using traditional Birth Attendant services; low female literacy level; delayed access to transport; hardship of long distance and physical terrain; delayed prompt quality emergency obstetric care; and delayed care while at the hospital due to patient refusal or concealment were observed. According to the 3Ds, the most common delay observed was in receiving treatment upon reaching the facility due to referral delays, missed diagnoses, lack of blood, lack of drugs, or inadequate care, and severe mismanagement.

## Introduction

In 2010, the global maternal mortality ratio (MMR) was estimated to be 210 per 100 000 live births, which translated into 287 000 maternal deaths worldwide [1]. In 2008, an estimated 358 000 women died worldwide due to pregnancy-related complications, with low-income countries accounting for 99% (284 000) of these deaths [2]. The Sub-Saharan Africa region alone accounted for nearly three fifths of these pregnancy-related deaths (162 000). In the same year, 3 000 women and girls died in Malawi. A woman living in Malawi has a 1 in 36 lifetime chance of dying during pregnancy or childbirth; while a woman living in an industrialized country, such as Norway, has a 1 in 7,900 risk. Though the maternal mortality ratio decreased from 1990 to 2010 (1 100 to 675 per 100 000 live births, respectively), Malawi has conceded that reaching the Millennium Development Goal (MDG) 5 target of 155 maternal deaths per 100 000 live births by 2015 is unlikely [3], [4].

Epidemiologically speaking, these numbers are very useful not only for determining the distribution, causes and risks of maternal death but also for promoting and protecting women and children’s health [5]. At the same time, these numbers tend to distance and detract us from the realities of those affected. Nevertheless, they are an essential piece of the maternal death puzzle.

The actual causes of maternal deaths such as hemorrhage, infection, pre-eclampsia, obstructed labour, HIV/AIDS, and abortion are also essential pieces of the puzzle. At the same time they are end-points of long, disjointed stories. If reducing maternal deaths were as simple as crunching numbers and implementing biomedical interventions to prevent them, all low-income countries, like Malawi, would reach MDG 5. However, because this is not the case we must go beyond the numbers and diagnostic classifications (International Classification of Diseases, 10^th^ Revision– ICD-10) to discover the underlying reasons why so many mothers continue to die.

To help explain why, information regarding the events and circumstances surrounding the deaths in question is needed. The maternal death audit is an approach used to gather this needed information in a systematic manner [6]. For low-income facility-based reviews are generally easiest to promote and implement because they do not require external expertise, advanced academic rigor or sophisticated questionnaires [7]. A facility-based maternal death review is defined as a qualitative, in-depth investigation of the causes of and circumstances surrounding maternal deaths occurring at health facilities [8]. The review process generates a massive amount of information that must be synthesized in a coherent manner while accounting for community and facility factors that contribute to maternal deaths.

The Three Delays conceptual framework is a comprehensive approach in connecting the “pieces,” i.e. the events and stories. Thaddeus and Maine assert that in most cases if prompt, adequate treatment is provided, adverse outcomes can be avoided [9]. Moreover, they propose that pregnancy- related mortality is overwhelmingly contributed to delays in three phases:

1. Deciding to seek appropriate medical help for an obstetric emergency which is influenced by the actors involved in decision-making; sociocultural factors; distance from the health facility; financial and opportunity costs
2. Reaching an appropriate obstetric facility, which depends on how far away the nearest facility is from her home in terms of distance and travel time; availability and cost of transportation; road conditions
3. Receiving adequate care when a facility is reached; factors affecting the receipt and provision of care (includes the adequacy of the referral system); shortages of supplies, equipment, and trained personnel; competence of available personnel, ineffective communication, and poor patient management

Though the interplay among these three phases is complex, they are not necessarily interdependent on each other. A delay in one phase may or may not exacerbate or prolong a delay in another. However, there is usually a combination of factors across the three phases that culminate in the woman’s death.

By putting the pieces together, we gain a better understanding of what actually happened. This is particularly true for cases in which women have either died en route to or upon arrival at the hospital, or when things have gone completely awry within the hospital itself. By gleaning clues from the various phases we can assist health management with adapting or developing interventions that improve the healthcare delivery system and the quality of care rendered. Furthermore, there is also a potential for empowering healthcare workers to examine their current practices and for communities to modify some of their cultural beliefs and behaviors.

The objective of this study was to identify facility and community based factors that contributed to maternal deaths and to formulate and prioritize some practical recommendations based on the Three Delays Model. The findings will assist researchers and program managers in determining complex factors associated with maternal deaths. The findings will also potentially help policy makers prioritize funding and maternal health care personnel modify their practices.

## Materials and Methods

### Ethics Statement

This study was carried out in compliance with the Helsinki Declaration. Ethical approval was granted by The College of Medicine Research Ethics Committee in Malawi (Proposal No. 10/08/703) and The Regional Committee for Medical and Health Research Ethics in South-Eastern Norway (2008/16105). In addition, permission to conduct the study was obtained from the District Health Officer, District Nursing Officer, director of the hospital; senior head nurses at both sites, and the village headman or chief in the respective communities were informed.

### Study Design

A descriptive retrospective case study design was used and qualitative methods selected to conduct an in-depth investigation and analysis of the circumstances and events surrounding individual cases of maternal deaths.

### Study Country

Malawi is one of the poorest countries in the world, both in terms of income and human development. The total expenditure on health was 4.8% of its Gross National Product in 2009 (ranking 142 out 190 countries) [10]. In 2010 it ranked 153 out of 169 countries with comparable data accessible to the UN in the Human Development Index [11]. It has a predominantly agricultural economy, based mainly on tobacco grown in the central region, sugar and tea in the southern region and timber in the northern region. With most of the population highly dependent on rain-fed subsistence farming, there is widespread food insecurity, as well as rampant poverty. For the 2000–2009 period, 74% of Malawians lived below the international poverty line of US$1.25 per day [12].

In Malawi maternal healthcare services are provided informally through traditional birth attendants (approximately 5000 practicing, of which 2000 have been trained by the government [13]). Formally, they are provided by midwives, nurse-midwives, clinical officers, general medical doctors, and gynaecologists/obstetricians. The provision of healthcare occurs at three different levels (primary, secondary, and tertiary) that are linked by a referral system. All maternity-related services are offered free of charge in government facilities and in some non-governmental facilities. At the primary level, maternal services are managed by nurse midwives who manage only normal deliveries, except for a few facilities that conduct vacuum extraction. Most Christian Health Association of Malawi (CHAM) hospitals and district hospitals in the public sector provide emergency obstetric care (EmOC) includes the administration of parenteral antibiotics, oxytocic drugs and anticonvulsants, as well as the manual removal of the placenta, the removal of retained products, assisted vaginal delivery, surgery (cesarean sections) and blood transfusion. Facilities that provide the first six are called Basic EmOC facilities, while others performing all eight signal functions are called Comprehensive EmOC facilities.

According to the World Health Statistics 2011, there were 3896 nursing and midwifery personnel, and 257 physicians in Malawi for the period of 2000–2009 [14]. Respectively, this represented a density estimate of 3 and less than 0.5 per 10 000 population. Malawi has one doctor per 62 000 population and vacancies among obstetrician–gynecologists, pediatricians, surgeons and other medical specialists range between 71–100% [15]. Vacancies among nurses stand at 65%. Another challenge is the uneven distribution of the workforce. Of the 190 Physicians who participated in the 2007 health personnel census, 62%work in urban areas, 23% work in rural areas and 15% work in semi-urban. Of the 2932 nurses/midwives who participated, 38% work in the urban areas, 33% work in rural areas while 29% work in semi-urban areas. Conversely, nurse technicians and medical assistants work predominantly in the rural areas (40% and 68%, respectively).

### Study Setting

The study was primarily conducted at two urban comprehensive emergency obstetric care (CEmOC) units of a secondary and tertiary hospital approximately five kilometers apart in Lilongwe, Malawi, with clinicians shared between the two sites. Together they have a catchment area of approximately 4 million inhabitants. The secondary hospital services non-paying patients, while the tertiary hospital has a mix of paying and non-paying clients, as well as referred clients from within and outside the district. Together, the maternal death numbers are estimated to be one per week, with a range of between two and six per month. Other characteristics of the facilities are provided in Table 1.

**Table 1:** Comparative characteristics of secondary and tertiary maternity units.

Characteristics	Secondary	Tertiary
Bed Capacity	220	220
Average number of admissions per month	1420	270
Average number of deliveries per month	1117	250
Average number of deliveries per day (24 hrs)	40	9
Total number of nurses in labor ward	23	12
Average number of nurses per day shift	7	4
Average number of nurses per night shift	7	4
Average number of nurses per day shift (wkend)	7	4
Average number of nurses per night shift (wkend)	7	4

### Study Participants

For the time period January 1, 2011 and June 30, 2011, women who died while pregnant or within six weeks of being pregnant, had received care or delivered at either hospital and resided in Lilongwe District prior to their death were included in the study. Specifically, the medical charts of deceased women, healthcare workers who provided care to the deceased women, family members along with guardians of the deceased and traditional birth attendants were purposively sampled.

The authors decided that a sample size of 20 to 25 cases was acceptable in terms of generating a comprehensive assessment of the contributing factors leading to the maternal deaths, as this range aligned with those observed in the literature. Guest, Bunce and Johnson identified seven sources that provided guidelines on actual sample sizes which ranged from five to 60 [16]. In Mason’s research in which he reviewed the abstracts of doctoral theses relating to interview-based qualitative studies in Great Britain and Ireland, he observed that sample sizes ranged from one to 95 [17].

The actual sample sizes were 32 maternal death charts, 34 healthcare workers, and 27 family/community members. The sample sizes allowed us to reach the saturation point. The principle of ‘saturation’ implies that the data collection is completed when no new insights or concepts emerge and/or when available participants have been exhausted [18]. For this study the saturation point occurred when respondents began repeating similar issues they or their deceased loved ones faced during the course of the complication to death.

### Data Collection

Data collection involved three activities: chart extractions, facility-based interviews, and community-based interviews using three different data collection tools adapted from the WHO guidelines: “Beyond the numbers: reviewing maternal deaths and complications to make pregnancy safer” [8]. More specifically, the following tools were used:

- **The Medical Record Extraction Form** was used to extract key information about each woman such as parity, gravidity, medical history, and antenatal visits. It was also used to record the facility’s classification of the death, to assess contributing factors that have been defined as delays, lack of resources and a lack of personnel. Senior management at the study hospitals provided the medical charts. The first author and two nurse/midwife technicians completed the form for each maternal death included in the study.
- **The Facility Staff Interview Questionnaire** was used to conduct interviews with healthcare staff who provided care to the deceased. It included open-ended, semi-structured questions to document healthcare workers’ accounts of the death in question, the actions taken, challenges with providing care, and avoidable factors. Respondents were identified by signatures the medical chart and schedule rosters. The interviews were conducted by the first author and the two aforementioned research assistants at a time and place convenient for the healthcare worker. The majority of the interviews were recorded (28 out of 34 interviews). For those that were not, detailed notes were taken. In some instances follow-up interviews and calls were made to clarify or elaborate on what was said.
- The maternal deaths that were included in the study were traced to their respective communities where family and community members, including traditional birth attendants when appropriate, were interviewed. **The Verbal Autopsy and Contributing Factors Questionnaire** was used. It served as a guide that evolved in an iterative, flexible manner, and was dependent on responses from the interviewees. It contained the following five sections: 1) Background–pertaining mostly to demographics of the deceased, her husband and child; 2) Symptoms observed or the woman herself experienced and voiced concern; 3) Existing Diseases a list of diseases that may have indirectly caused the maternal death (or adversely impacted the pregnancy) 4) Health seeking behaviour/contributing factors–to assess what the woman and those caring for her did between the time she fell ill and her death; and 5) Family’s account of events surrounding the woman’s death and illness. The interviews were conducted in the participants’ homes and took place within two to nine months of the maternal death. Like the first two activities, the interviews were conducted by the first author and the two research assistants. Most of the interviews were recorded (26 out of 27 interviews) whereas detailed notes were taken for the one that was not recorded. Lastly, follow-up interviews and calls were made to help clarify or elaborate on what was said.

### Data Analysis

The data that were extracted from the medical charts (e.g. the age of woman at death, the gestation weeks, the number of years of education, and amount of time elapsed between pregnancy and death) were analyzed using Predictive Analytics Software Statistics 18.0 (PASW, formerly known as SPSS Statistics). The analysis was descriptive in nature.

The transcripts from both the Facility Staff Interview Questionnaire and the Verbal Autopsy and Contributing Factors Questionnaire were analyzed using a directed approach to content analysis [19]. This approach was used because the Three Delays Model and existing maternal death research helped determine the initial coding scheme and relationships between the codes which Mayring has referred to this as deductive category application [20]. The transcripts were read carefully to form a general impression of what healthcare staff and family members said about the respective maternal death cases. The transcripts were then re-read to understand the context in which the maternal deaths occurred. Based on the definitions of the three phases of delay, all text that appeared to describe any of the delays were highlighted. Through the deductive category application all highlighted text was compared and sorted according to the predetermined categories of delays in deciding to seek care, reaching a facility and receiving care once a facility was reached. For each category, the data was reexamined to determine whether subcategories were needed. Text that could not be coded into one of these categories was coded with another label, which led to the addition of one new category.

For each maternal death case, the data from the respective medical chart and interview transcripts were triangulated to gain a more accurate account of what transpired. Based on the International Classification of Diseases tenth version (ICD-10), clinical judgment and experience, two gynecologists/obstetricians independently reviewed the triangulated data for each maternal death and determined the causes of deaths. They either confirmed the documented causes of deaths or provided alternative causes. Where appropriate, they also provided insights on what could have been done differently to potentially prevent the deaths. One of the gynecologist/obstetricians has worked in the study sites since 2004. He was instrumental in establishing the Norwegian-Physician Exchange Program (Norwegian doctors provided technical assistance, worked in the maternity ward for a 6-month rotation). The other gynecologist/obstetrician has over 20 years of experience working in various countries in Africa, including Malawi.

## Results

The findings are divided into two sections. The first presents the numbers and causes of maternal deaths. In the second section, themes of the underlying contributing factors are provided. In addition, a summary table with the contributing factors categorized according to the Three Delays Model is provided.

### First Piece of the Puzzle – The Numbers

#### Facility statistics

According to the maternity records, there were 58 maternal deaths and 8108 live births recorded during the period of January 1 – June 30, 2011. This translates into an institutional maternal mortality ratio of 715 per 100 000 live births (i.e. 58/8108 × 100 000). The demographics section below provides more details about the actual number of cases that were reviewed (32 of the 39 eligible cases).

#### Demographics

Forty-eight maternal deaths occurred at the tertiary hospital during January 1– June 30, 2011. Of them 29 had lived in Lilongwe District, while the other 19 had resided outside of Lilongwe District prior to their death and therefore not reviewed any further. Fourteen of these 29 were referred from the participating secondary hospital. We were able to locate and review medical charts for 24 of the 29 cases. For the same time period, 10 maternal deaths occurred at the secondary hospital. All had lived in Lilongwe District. However, two charts were missing. Therefore, eight of the 10 maternal deaths that were recorded at the secondary hospital had a medical chart for us to review.

A total of 32 maternal death cases were reviewed (24 from the tertiary hospital and eight from the secondary hospital). The cases were from 21 different areas within Lilongwe District. The deceased women had an average age of 28 (range = 16–39, SD = 6.7). On average they were pregnant three times (range = 1–10, SD = 2.4) and had given birth twice (range = 0–9, SD = 2.4) and had two antenatal care visits for their last pregnancy (range = 0–5, SD = 1.5). Thirteen of the women were HIV positive and nine were HIV negative while the HIV status of the remaining 10 was unknown. For the 19 women for whom we had the number of years of schooling, the level of formal education varied, ranging from three years to 14 years, SD = 3.7. Only six of the 19 completed secondary school. For the 18 for whom we have work information, nine were housewives (with four of them being subsistence farmers), one was a cook, one a cleaner, five were self-employed (selling clothes, firewood and produce), and two were nurse/midwife technicians.

#### Stage in pregnancy upon death

The deaths were grouped into seven categories based on pregnancy stages (Table 2). Over one third of the women died between one and 42 days after delivery (12 out of 32 cases). For these 12 deaths, the average number of days between delivery and death was 11 (range = 1–41). Ten women died at less than 28 weeks gestation age. The average age of gestation was 18 weeks (range = 12–25). The third largest category in which maternal deaths fell was dying within 24 hours of delivery. There were eight cases, dying with the average number of six hours (range = 17 minutes –12.75 hours). Finally, two died during labor. The majority (29 out of 32) died inside the institutions, with four dying immediately upon arrival. The remaining three died en route to the hospital including two who died while being transported to the referral hospital.

**Table 2:** Stage of Pregnancy upon death.

	Frequency
<28**wks	10
>28**wks	0
Intrapartum Stage I*	2
Stage II	0
Postpartum up to 2**hrs	3
Postpartum 2 to 24**hrs	5
1 to 42 days	12
Total	32

* In labour, prior to completion of the second stage.

The 32 maternal deaths were reviewed and categorized according to their respective antepartum, intrapartum, and postpartum state upon death.

#### Causes of death

Based on clinical diagnoses, completed maternal death audit forms, and facility interviews, 16 cases resulted from direct obstetric complications. The majority of maternal deaths in this category were due to sepsis and hemorrhage (six each). Sixteen of the maternal deaths were due to indirect causes with the main cause being anemia (5), followed by heart disease (4) and HIV (3). Table 3 provides a detailed enumeration of the causes of deaths.

**Table 3:** Direct & Indirect Causes and Frequencies of Maternal Deaths.

Cause of Death		Frequency
Direct	Sepsis	6
	Hemorrhage	6
	Hypertensive disorder	3
	Obstructed labor	1
Indirect	Anemia	5
	Cardiac disease	4
	HIV-related disease	3
	Malaria	2
	Hepatic failure	1
	Renal failure	1
Total		32

Based on chart notes and two independent reviews, the cause of death for each maternal death was determined, confirmed, tallied, and grouped according to whether the cause contributed directly or indirectly to the death.

### Second Piece of the Puzzle – The Underlying Factors

Based on Thaddeus and Maine’s Three Delays Model, contributing factors specific to each maternal death investigated were categorized according to the corresponding phases of delay and are presented in Table 4.

**Table 4:** Summary of Maternal Death Characteristics and factors contributing to the 3 Phases of Delay.

Case	GP^i^	Age	Yrs of School	No.ANC^ii^ visits	HIV Statusiii	Cause of Death	Delay 1^iv^	Delay2^v^	Delay3^vi^	Comment
01	G2P1	22	6	2	+	Cardiac arrest, Malaria & Anemia	severely anemic w malaria & infection didn’t seek health care	–	ANC visits didn't catch malaria, or admit her to high risk antenatal ward for anemia, only given 1 pint of when Hb was 2.3 when admitted	first ANC visit she was told she was anemic, same day at home she exp heart palpitations, following day went to the hosp and was admitted
02	G3P0+1	21	5	0	–	Valvular Heart Disease	Experienced chest pain, heart palpitatations and shortness of breath for 1 month	–	–	–
03	G1P0	27	12	UNK	–	Puerperal sepsis	–	–	missed the tumor during previous visit, delay in referring approx 2 days due foul smelling discharge/necrotic uterus	skilled staff to do hysterectomy weren't avail, had to wait till baby was better before referring her, the mother didn't receive immediate attention
04	G2P2	24	12	5	+	Peritonitis postpartum	home delivery	–	missed placental lobes the following day at the HC, operation 3 times, still gaping hole, became infected	–
05	G1P2	33	12	UNK	–	Pre-eclampsia	–	–	2^nd^ admission they didn't do anything: no blood for severe anemia, no meds for BP or antibiotics for pneumonia; discharged to postnatal ward too early	–
06	G3P2+1	29	10	4	UNK	Renal failure due to puerperal sepsis	stayed home for 5 days w/o urinating or defecating	–	given wrong medication in renal failure, urgent lap wasn't done, x-ray not done-no radiographer, discharged early on 1st admission	–
07	G2P2	34	14	2	+	Lactic acidosis (HIV-related)	had abd pains for 1.5 months	–	she wanted to terminate the baby but prohibited at hospital, was referred; concealed HIV status, didn’t take ARTs for 1 month refused to be tested delayed correct Rx	she was a nurse midwife technician, went to Likuni to check the file, but pages were missing
08	G3P2	35	12	0	+	cryptococcal meningitis	exp headache for almost 1 month	–	tried lumbar puncture but not successful	sister believes that if she had started on ART early that could have boosted her immunity and saved her
09	G1P1	18	9	4	–	Heart failure pulmonary embolism	–	–	Waited 1.5**hrs before being reviewed by clinician	–
10	G4P3+1	39	14	2	+	Hypoxia, eclampsia	–	–	family requested referral letter which was delayed, CO had dilemma with when to refer, out of BP med but kept her & asked husband to go buy med	they didn’t have the BP med in the first place should have referred immediately
11	G7P6+1	32	5	2	–	Concealed ruptured uterus due to obstructed labor	tried to deliver at TBA, husband was contacted late Husband gave her transport money but she preferred the TBA	took husband an hr to find transport	–	–
12	G5P5+1	40	8	1	UNK	PPH	–	1^st^ got a bike, but she couldn't ride it so had to find a cart. It was dark she delivered twins on the way, the ox went their separate ways, TBA was contacted to deliver the placenta, she bled out	–	–
13	G2P1	28	10	4	–	Intrapartum hemorrhage due to ruptured uterus	–	–	request for C/S denied, discounted; she was not monitored	they didn’t take her cries seriously, the clinician didn’t see her at all
14	G2P1	22	8	2	–	Valvular Heart Disease - Stenosis	preexisting heart condition, didn't comply with treatment or advice	–	ambulance from hosp to HC delayed 2**hrs	–
15	G6P5+1	32	3	2	–	PPH	delivered at TBA	had to get a cart, went home to pick up health passport before going to HC	while in ambulance IV line came out while being transported to referral hosp	–
16	G1P1	20	8	2	UNK	PPH	delivered at TBA, over stayed	–	–	–
17	G2P1	20	UNK	2	+	Anemia	?	?	for 3 hrs being managed in the wrong ward, in gynae instead of HDU on admission, lacked blood which was ordered right away, she didn't even receive 1 pint	nurse thinks she should have gone directly to ICU
18	G4P2	35	UNK	3	+	PPH	?	?	no blood, was only given 1 adult & 1 child pint, needed at least 4 pints	Because was Hb 2.2 referring hosp should have given her blood or mobilized to get some
19	G1P0	18	UNK	0	+	Bacterial meningitis	had symptoms for 2 days headache, vomiting, and diarrhea 7xs	?	not informed during handover, patient not seen/reviewed by clinician 4–8 referring HC didn't do a thorough history assessment preg or HIV test not done	–
20	G5P5	35	UNK	UNK	UNK	Anemia	had exp dizziness, chest pain and very pale for a few days	?	transferred to postnatal instead of HDU, referral letter had no details about what was done and what needed to be done	the nurse working was on another bed and then the guardian told her that the patient had died
21	G1P0	30	UNK	UNK	UNK	Severe Anemia	?	?	Out of her blood type (B+)	–
22	G10P9	39	UNK	UNK	UNK	Peritonitis postpartum	?	?	classical incision was necrotized	–
23	G2P1+1	31	UNK	UNK	+	Hepatic failure	?	?	?	the family went to a secondary hosp instead of coming directly to tertiary hospital they ended up being referred which took more time
24	G1P0	24	UNK	4	UNK	Eclampsia	?	?	?	referral time from one place to another was not documented
25	G1P1	16	3	0	UNK	Anemia	?	?	At 5 months admitted to Gyn ward and transfused but discharged after having Hb 5; referred w Hb 1.2 but hadn't given her blood	–
26	G3P1+1	33	UNK	UNK	+	Anemia	hadn't felt fetal movement for a month, while in the hosp she didn’t receive ART	?	interventions planned/written down but not completed, been there 12 days and not transfused	–
27	G6P5+1	37	8	2	–	PPH	–	–	patient refused blood because she's Jehovah’s witness	–
28	G3P2+1	25	UNK	UNK	UNK	Septic abortion	?	?		we don't know the time she was referred from the HC to 1^st^ hosp to 2^nd^ hosp
29	G2P1	25	UNK	2	+	septicemia post delivery	?	?	Referred w/o treating her for sepsis, clinician late w reviewing, lab not able to do CD4 count at night	she was in Stage III supposed to be on ARV but was just on cotrimoxazole
30	G4P2+1	32	UNK	2	+	lactic acidosis (HIV-related)	?	?	?	KCH provided good services, mgmt to the patient
31	G3P2	23	UNK	UNK	+	Sepsis & induced abortion	?	?	Due to lying, was treated for malaria, couple of days later admitted to attempting abortion	before leaving hosp XX, while being referred, she collapsed and died
32	G10P7+2	35	1	2	–	Cardiac arrest, severe pre-eclampsia	Had no functioning of one side for 6 days, the evening before going to the hospital she could not walk	–	–	Through faith she thought her condition would get better. The husband didn’t think there was anything he could have done to save her.

i Gravida, Parity

ii Antenatal care

iii + positive, - negative, UNK unknown

iv Delay 1 Delay in deciding to seek care

v Delay 2 Delay in reaching a health facility

vi Delay 3 Delay in the provision of care

Through chart reviews and interviews with the families, friends, TBAs and healthcare providers, we gathered demographic and clinical information, and information on both socio-cultural and facility factors that may have contributed to the respective deaths. These factors were categorized according to the appropriate delay.

### Delay in Deciding to Seek Care (Phase 1 Delay)

The delay was determined from the moment the woman, a family member or someone else realized that there was a complication until the decision to seek care was made. Several factors influenced the decision to seek care by the women who died. In the current study the main factor was recognizing the signs. Additionally, using traditional birth attendants also played a part in delaying the women.

### Recognizing Signs, Symptoms, and Severity

Obtaining medical care for women with obstetric complications begins with the local recognition of warning signs and symptoms [9]. Eleven of the 32 women had illnesses and symptoms from two days up to a month before they or their families decided to seek care at a hospital. They tended to underestimate the severity of the problem as in the case where one woman lost the use of one side of her body for six days. Her husband stated that they delayed because neither of them thought it was serious, and that through faith she would get better.

### Traditional Birth Attendants and Home Delivery

Another factor that delayed the deceased women in receiving appropriate care was seeking assistance from a traditional birth attendant (TBA) or delivering at home instead of deciding to go directly to a health facility. This occurred in four of the cases. Two of the four women made the decision themselves to go to the TBAs’ respective places for delivery, as narrated by the neighbor and husband of one of the cases.

Case #11 Respondents: neighbor/guardian & husband:

*Labor started during the day but she wanted to go late in the afternoon because she was afraid of the neighbor seeing her. …she asked me to go with her to the TBA. We walked and got to the TBA at 7 pm. The TBA was able to examine her wearing gloves. Then she was told to push. Then she was examined for the second time around 4 am when the TBA could see that the baby was not coming out. She was weak. The legs and the knees were cold so that they stopped functioning. Then the TBA phoned the husband and told him about the situation so that he could start looking for a car to hire, but it was during the early hours. This is a new area [where the deceased woman and husband lived] so it was difficult to find transport. The minibuses refused because they don’t want to help people in labor. It was up to 8:00 am before the husband could hire the car to collect her to the hospital. When she was in the car she could not talk, she could not even drink anything. We were unable to communicate with her for the whole ride to the hospital, 9:00 am. When we got to the hospital, the nurses said, 'you have brought us a dead person.'*

In the other two cases the TBAs were summoned to assist on location due to remoteness from a health facility or urgency of need.

### Delay in Reaching the Health Facility (Phase 2 Delay)

When the hospital is far away, the mode of transport becomes an important determinant in how soon treatment will be received and consequently in whether the woman and or child survives [21].

#### Access to transport

For three of the 17 families with whom we conducted verbal autopsies, transportation was an obstacle. One family lived in a densely populated area near the hospital. However, the husband still had problems locating transportation in a timely manner due to the fact that the emergency occurring at the break of dawn (narration shared above, Case #11).

### Hardship of Long Distance and Physical Terrain

The other two cases lived in rural areas where they only had access to animal carts. These two women died en route to the hospital, while the former died upon arrival at the hospital. All three had difficulty locating transport in the middle of night which further delayed the women in receiving treatment. The two narrations below provide vivid accounts of the hardships faced by the women and their families while attempting to reach the nearest facilities.

Case #12 Respondents: Mother and younger sister:

*She looked normal at first. Towards the afternoon around 1 pm she started complaining that she was not well. So we got prepared to go to the hospital. We are far away. So to get a bicycle, to find someone to carry her (was difficult). We got a cart because she could not manage on a bike. So we got the cart when it was late in the day, around 5 pm. So they (the deceased and her husband) went in the cart and this one (pointing to the deceased’s younger sister) went with her. On the way at Pambala, there’s a small bridge under a gum tree, that’s where she delivered (twins). They say that’s where she delivered. It was very dark; they didn’t even have a torch. They had to rush to the nearby houses to borrow a torch. Then they saw that she was not well. Immediately the ox left and ran back home. So what are we going to do? …The ox ran away, so they had to look for other ox from other people. And after a little while she died at the same spot. So they had to harness the ox on the cart and return home. So he (the deceased woman’s uncle) said, ‘when you get home a car will come early in the morning to take the body to the mortuary.*

Case #14 Respondents: Mother, father and aunt:

*It’s (the health facility) too far. There are hills and valleys on the way so the guy with the bike some times had to get off the bike and start pushing the bike while the patient was sitting on the bike, so it took time. For instance on this last trip we left around 6 o’clock we got to XX around 8 o’clock; it’s hilly. When they received us, they checked in the health passport book and explained to us that they would not do anything because this was a serious case. ‘We must refer you to X,’ so they really tried calling back and forth. They didn’t move from where we were; they really helped us. It was the ambulance that took time to arrive. We waited for a long time. The ambulance picked us up after 12 midnight. We got to X (according to clinical officer at 2:40 am) and they received and assisted us well, but it was her heart.*

### Delay in Receiving Quality Emergency Obstetric Care (Phase 3 Delay)

Even when the women reached the facilities, they did not receive adequate treatment in a timely manner or any treatment at all. Over two thirds of the women in the study experienced some type of treatment delay. Specifically, shortages of skilled staff, inadequate clinical work ups (history taking and documentation), missed diagnoses, insufficient communication among staff and to families, the denial of technical skill limitations, a lack of monitoring and attentiveness, a lack of blood, and a lack of hydralazine were all reported. In the case below, several of the aforementioned factors were at play.

Case #13 Respondents: 2 nurse midwife technicians (NMT) & husband:

*The woman arrived in the labor ward during the evening with strong contractions, 1 previous scar with normal fetal heart rate but she was screaming with pain and advising us on duty, especially me myself, that she would be better if she went for a cesarean section. But because everything was normal for her, I just counseled her that she shouldn’t be worried. Around past 9 pm I gave handover to a nurse midwife technician to continue taking care of the patient since I was going for a rest for four hours and later after coming from a rest to be told that the patient had died. …I had hoped that she was going to deliver vaginally without any problem. Going for a lie was a problem because my colleagues who received the handover were very few to look after all the patients in labor, including the patient I was handing over… From what my colleagues told me, they said that the theater was so busy through the night. This mother was supposed to be monitored very closely, although my colleagues told me that she was monitored. My assumption is that if she ruptured in the labor ward this means that it took some time to be noted otherwise they could have noted in the first place then probably rushed with her to caesarean section as an emergency. (Nurse-Midwife Technician #2)*

### Delay in Receiving Care in the Hospital due to the Patient (Phase 3B Delay)

When three of the women reached the facilities, they did not receive adequate treatment in a timely manner or any treatment at all due to concealing pertinent information and due to religion. This occurred in cases #7, #27, and #31.

#### Concealment

In case #7 the deceased woman was HIV positive, but because she worked as a nurse where she was also a patient she did not want her colleagues to know her status. Out of the 13 cases with a known HIV positive status, she was the only one who deliberately concealed it from medical staff. They saw how ill she was and insisted that she take an HIV test so she could be treated appropriately but she refused up until she was referred to the tertiary hospital. During this concealment period, which was almost a month, she was not on antiretroviral therapy.

In case #31, the young lady who was covered in blood was found by a distant relative. The relative took her to the hospital the same day but she lied about being pregnant so the out patient department gave her antimalarial drugs instead of referring her to the gynecology ward. Two days later she returned and admitted to being amenorrheic for four months and attempting to induce an abortion.

### Religion

Case #27 made it to the hospital in a timely manner and blood was in fact available, but because of her being, Jehovah’s Witness, she refused to be transfused.

## Discussion

The findings illustrate that multiple factors conspired to delay pregnant Malawians from receiving appropriate obstetric care while simultaneously conspiring to delay healthcare facilities from providing this much needed care. The Three Delays Model helped us piece together various factors and contextualize them in the larger picture of maternal mortality in Malawi.

### Delay in Deciding to Seek Care (Phase 1 Delay)

Failing to recognize the gravity of the situation which resulted in delaying the decision to seek care until it was almost too late to provide the urgently needed care has been observed in other studies [22]–[24]. With pregnancy and childbirth being natural processes that take place in a specific cultural context, a possible explanation for this delay may be that some of the women might have dismissed certain signs and symptoms as being a normal part of the journey into motherhood [25]. In a study among pregnant women in Senegal, 13% regarded fever, pallor and dizziness as normal signs of pregnancy because these conditions were common among pregnant women in that area [28]. In Tanzania, rural women seemed to avoid going to the hospital due to different interpretations of danger signs [29]. Another explanation to the delay may have been due in part to relying solely on one’s faith as observed in one of our cases and also by Cham and colleagues [26]. Jansen asserted that religion, medicine and magic are closely interwoven [34]. If the barriers to care are too overwhelming, a culturally based reassurance that “things most likely will go well” may cause a hesitation in recognizing early signs of complications.

### TBAs

We speculated that for a couple of the deceased women they might have preferred to deliver at the TBA because they would receive one-on-one care that is closer to their homes than going to an unfamiliar, distant hospital [13], [27]–[29]. For the other two it was a matter of having no other choice, as observed in other studies [27], [30], [31]. In Malawi there are approximately 5000 TBAs and 2000 of them have been trained by the government of Malawi since 1976 [13], [28]. Although they are not considered a part of the formal healthcare system, they fill a gap of uneven distribution of healthcare personnel, particularly in the rural areas. According to the latest Demographic and Health Survey for Malawi, almost 25% of births, in the five years preceding the survey, took place at home. These were most likely with assistance from a TBA [4]. For Lilongwe District, where the current study took place, 28% of births took place at home. TBAs are highly esteemed in their respective communities. However, that is not enough when an obstetric complication arises.

The government of Malawi has adopted the WHO recommendation of redefining the roles of TBAs [32], [33]. However, as of yet there is no strategic/action plan to effectively operationalize and enforce adherence to the TBAs’ redefined role. Research on TBAs’ new role and effects on delays and maternal mortality reduction is warranted.

### Delay in Reaching the Health Facility (Phase 2 Delay)

Delays such as these have been reported in other studies [23], [26]. The main reason why we did not observe more cases facing this challenge is perhaps because the catchment area for the hospitals is semiurban with very good, accessible roads to the hospital. Nevertheless, the physical distance and difficulty with locating transport faced by the two rural cases connote aspects of remoteness such as poor road infrastructure, poor communication between communities, poverty, limited access to information, and strong adherence to traditional values [34]. Solutions must be devised that either bring the services closer to the women or make public transport readily available and affordable. Another low-cost alternative that is becoming more popular and gaining more interest in Malawi, and other low-income countries, are maternity waiting homes (MWH).

MWHs are residential facilities, located near a qualified medical facility, where women defined as “high risk” can await their delivery and be transferred to a nearby medical facility shortly before delivery, or earlier should complications arise [35]. They are considered to be a key element of a strategy to “bridge the geographical gap” in obstetric care between rural areas, with poor access to equipped facilities, and urban areas where the services are available. However, findings from a systematized review of the effectiveness of MWHs on improving maternal and neonatal outcomes in low-resource countries were inconclusive [36]. There was insufficient evidence upon which to base recommendations for practice (i.e. to promote the wide use/scale up of MWHs).

### Delay in Receiving Quality Emergency Obstetric (Phase 3 Delay)

This third delay is typically indicative of suboptimal quality of care [9], [37]. The aforementioned factors have led to inadequate monitoring, missed and incorrect diagnoses, delayed or incorrect treatment, delayed referrals and transfers, patients not being stabilized before referring, premature discharges, and outright negligence. Our findings were similar to those observed in other studies [24], [26], [38]–[40]. Two of these studies were conducted in Malawi. The findings illustrate symptoms of a weakened, under resourced healthcare system riddled with deficiencies and malfunctions. As noted in other studies, the healthcare delivery system failures identified in our study contributed to these women’s deaths [21], [26], [41]. In a recent (2012) study in which 40 articles, spanning 15 years, were reviewed, a shortage of resources, supplies, equipment qualified human resources including lack of specialized training were all identified as significant barriers to utilizing emergency care [42]. Cannoodt and colleagues also identified articles that reported deficiencies within the organization of services as contributing factors for low utilization. Specifically, a lack of accountability and proper management, as well as a lack of communication and referral systems were identified. If these failures are addressed, they may have a significant impact on the utilization of maternal health services and on the reduction of maternal deaths [37], [43]–[46].

### WHO Guidelines for Maternal Death Review Research

The data collection tools were useful in ascertaining in-depth information on clinical, sociocultural, and other contributory factors of the maternal deaths reviewed. They included a combination of open- and closed-ended questions which helped balance the requirement of participants to recall and recognize information. The questions helped participants, both relatives/community members and health care staff, narrate the facts that led to the maternal deaths. At the same time the question elicited the relatives’ perception of the conditions surrounding the death of the women by encouraging them to freely reconstruct the process that led to that event and to describe its most relevant aspects.

Conversely, however, there were some sections that were problematic. For example, on the facility staff interview questionnaire there were questions that asked about the participants’ knowledge of the purpose of antenatal visits (e.g. routine or problem), the women’s health status prior to becoming pregnant, and factors affecting the women’s condition prior to arrival. Antenatal visits and health status are typically recorded in the health passport which is kept by the woman herself. Thus, unless the health care worker has access to this passport, s/he is unlikely to be able to answer these questions. Similarly, unless questions regarding events occurring prior to arrival are asked and recorded during admission, s/he will also not know this information. Lastly, some of the questions related to avoidable factors seemed to lead participants to assume that something happened in the community, while blaming the women (e.g. coming late/transportation issues) without any concrete evidence.

Regarding the verbal autopsy questionnaire, a problem occurred when asking relatives to recap the events, as some participants could not recall the exact timing of events. The concept of time was difficult for them (none wore/owned a watch), which made it more difficult to construct a timeline of the events preceding the death. Moreover, because the mode of transport was usually by foot, the distance travelled from point A to B to C, and so forth was difficult to determine as well. These two factors also made it difficult to determine the degree to which the woman and her family experienced delays from the onset of complications to reaching the nearest facility, and from receiving care to death. Through triangulation of data sources (e.g. referral letters, admission registries, and health care worker interviews) we were able to fill in some of the gaps.

### Three Delays Model

Categorizing the contributing factors according to the Three Delays Model helped us determine where improvements could best be made to save lives in the future. Our study contributed to the model by adding another dimension to the third delay. Not only can the health care system and staff contribute to the delay in receiving care once in the hospital, but also the patient herself as observed in our study (Phase 3B Delay). Concealment and refusal by the patient had not been discussed in Thaddeus and Maine’s, or Gabrysch and Campbell’s delay models [9], [34] and may warrant further investigation.

Although the Three Delays Model is commonly used to explain why delays in accessing emergency obstetric care occurred, it does not address issues with accessing antenatal or postnatal care. The model assumes that delays are only linked to complications. The model does not take into account that in the absence of a complication a woman might still delay in seeking care. Moreover, indirect causes of maternal death, such as anemia in pregnancy, chronic infections, and HIV/AIDS, are not necessarily regarded as emergencies or complications however timely and appropriate management is required. Lastly, it does not provide the rich and detailed set of factors and interactions as illustrated through the access to health care conceptual model [47]. The conceptual model recognizes access as the outcome of a process involving the interplay between the characteristics of the health care service system and the potential users in a specified area, which is moderated by health care related public policy and planning efforts. Access has two dimensions spatial (geographic, space, distance) and aspatial (social, cultural, economic, or political). Consequently, the health services and those of the potential users can either be barriers or facilitators, which greatly influence the use of services.

### Conclusion and Recommendations

The Three Delays Model helped us gain a better understanding of why these women died. Not only did they die due to an obstetric pathology but also due, in part, to a complex web of delays stemming from the community and within the facility. The three delays conspired to form a downward spiral where the third delay represented a failure to seize the last opportunity to save the women [9], [37]. The Three Delays Model helped us put together critical pieces of the maternal death puzzle and contextualize them into the larger Malawian society. Our study revealed that the majority of the women who were poor, had low literacy and lived in semiurban areas, did in fact reached a health facility, but they did not receive the adequate or timely care needed to save their lives. Ideally we would like all delays in all phases to be addressed, but that is not realistic or feasible. Therefore we suggest prioritizing practical solutions for delays 3 and 1, and then lastly delay 2.

Instead of providing a detailed prescription of solutions, we briefly describe a comprehensive strategy that has been documented to reduce all three phases at varying degrees [48], [49]. It was developed by the Maternal and Neonatal Health (MNH) Program of The Johns Hopkins Program for International Education in Gynecology and Obstetrics (JHPIEGO). It is called the birth-preparedness and complication readiness (BP/CR) matrix. The overarching objective is to promote and improve the timely use of skilled maternal and neonatal care [50]. The premise underpinning the strategy is that preparing for birth and being ready for complications reduces all three phases of delays. BP/CR addresses these three delays in the continuum of pregnancy, labor and childbirth, and postpartum and newborn care at five different levels:

- The individual and family level - learn to recognize danger signs, identify a skilled provider and birth location, save money, identify potential blood donors, designate a decision maker, and arrange for transportation:
- The communities - like families, make arrangements to retain money, transport, or a blood donor to assist the women in reaching and receiving care in case of an obstetrical emergency:
- The health facility - ensure that the required equipment, supplies, and support systems are available:
- The provider - clinical personnel acquire or improve the necessary knowledge and skills needed to attend normal childbirth and manage obstetric and newborn complications; and
- The policymakers - institutionalize evidence-based healthcare policies and assure adequate funding for maternal and newborn healthcare services.

In conclusion, investing in family and community education, TBA role modifications and referrals, or improved transportation networks are futile when women actually reach the hospital only to die from inadequate care. The accessibility and provision of high quality emergency obstetric interventions are essential for improving maternal survival. The district health office and hospital administration must thoroughly and systematically investigate the healthcare delivery system malfunctions that are contributing to maternal deaths and devise urgent remedial solutions.
